# Pharmacotherapy for psychiatric inpatients with alcohol use disorder or acute intoxication: results from an observational pharmacovigilance program—status and changes between 2000 and 2016

**DOI:** 10.1007/s00702-025-03012-z

**Published:** 2025-09-04

**Authors:** Beatrice Haack, Johanna Engel, Philipp Pauwels, Sermin Toto, Stefan Bleich, Johanna Seifert, Renate Grohmann, Martin Heinze, Oliver Zolk, Phileas Proskynitopoulos, Timo Greiner, Michael Schneider

**Affiliations:** 1 University Department of Psychiatry and Psychotherapy, Brandenburg Medical School, Immanuel Klinik Rüdersdorf, 15562 Rüdersdorf, Germany; 2 Department of Psychiatry, Social Psychiatry and Psychotherapy, Hannover Medical School, 30625 Hannover, Germany; 3 Department of Psychiatry and Psychotherapy, LMU University Hospital, LMU Munich, 80336 Munich, Germany; 4 Institute of Clinical Pharmacology of the Brandenburg Medical School, Immanuel Klinik Rüdersdorf, 15562 Rüdersdorf, Germany

**Keywords:** Alcohol use disorder, Pharmacovigilance, Antipsychotic drug, Antidepressant drug, AMSP, Psychiatry, Psychopharmacology

## Abstract

Alcohol use disorder (AUD) is a mental disorder with a high prevalence and is one of the most common diagnoses requiring inpatient treatment. For the pharmacological management of withdrawal and detoxification, tranquilizing and anticonvulsant drugs, as well as symptom-triggered therapy, are recommended. In this study, we investigated the use of psychotropic drugs in the inpatient treatment of patients with AUD or acute intoxication by analyzing data from the *Drug Safety Program in Psychiatry* (German: Arzneimittelsicherheit in der Psychiatrie; AMSP). Patients were classified according to the ICD-10 code F10. The study included 10,332 patients treated for AUD or acute intoxication in participating hospitals between 2000 and 2016. The most frequently used drug classes were antidepressant (31.2%), antipsychotic (29.7%), anticonvulsant (26.4%) and tranquilizing drugs (24.3%), among which benzodiazepines were the most commonly used (23.6%). The most common drugs were carbamazepine (11.1%), diazepam (10.1%), mirtazapine (8.5%) and oxazepam (8.2%). Treatment patterns varied depending on sex and the presence of additional psychiatric diagnosis, such as depressive disorder. During the observation period, the use of antidepressant and antipsychotic drugs increased and the use of tranquilizing drugs doubled, while the use of clomethiazole declined. These findings underscore the need for greater attention to managing comorbidities. However, we also observed a high utilization of non-benzodiazepine sedating drugs—such as trazodone, mirtazapine and quetiapine—even though there is insufficient evidence to support their use in this context. Further research is warranted.

**Supplementary Information:**

The online version contains supplementary material available at 10.1007/s00702-025-03012-z.

## Background

The consumption of alcohol is widespread and an inherent part of our society. In Germany, 17.6% of the population engages in risky alcohol consumption and 3.4% are alcohol dependent (Rauschert et al. 2022). Every year, approximately 62,000 alcohol-related deaths occur, accounting for 10% of the total mortality in Germany (DHS Jahrbuch Sucht 2022 2022). However, only approximately 5% of those affected receive psychiatric or neurologic treatment; most are cared for by a general practitioner, whereas only a small proportion seek inpatient treatment (Zaudig and Trautmann 2006). Nevertheless, alcohol use disorder (AUD) has a high prevalence in inpatient treatment in Germany. With a total of around 293,000 inpatients, alcohol-related psychiatric and behavioral disorder ranked as the third most common ICD-10 diagnosis made and treated in inpatient stays in German hospitals in 2020 (Schaller et al. 2022).

More than half of patients with AUD suffer from at least one additional psychiatric diagnosis, such as depressive disorder (Zaudig and Trautmann 2006). In addition, long-term drinking can result in potentially life-threatening somatic sequelae such as gastrointestinal diseases, malnutrition, bone diseases, avitaminoses and neurologic diseases such as polyneuropathy and Wernicke-encephalopathies (Haber and Kortt 2021) (Engelhardt et al. 1985).

AUD treatment in Germany includes qualified detoxification, psychotherapy, psychoeducation, pharmacotherapy and support groups. While abstinence is the primary goal, controlled drinking may be more achievable for some. Relapses remain a common occurrence: 85% of patients experience relapse following initial detoxification without follow-up care (Batra et al. 2016), most within the first six months (Kiefer et al. 2022). On a more optimistic note, long-term treatment plans can reduce relapse rates by 50% (Heinz et al. 2003).

Several guidelines for the treatment of AUD are currently available (Kiefer et al. 2022; NICE UK 2011). The S2 guidelines for German-speaking countries relevant to a large part of our study period (i.e., 2000–2016) were published in 2003. It was first revised in 2014 and then followed by the S3 guidelines in 2020 (Kiefer et al. 2020) and its update in 2022 (Kiefer et al. 2022). Whereas clomethiazole was still recommended as the drug of choice for severe and moderate withdrawal symptoms in 2003 (Mundle et al. 2003), the newer versions primarily recommend benzodiazepines (Kiefer et al. 2022), although it is off-label use. Additionally, treatment for comorbid depression was still advocated in 2003 with tricyclic antidepressants (TCAs) or selective serotonin reuptake inhibitors (SSRIs), which are no longer mentioned in later reviews (Mundle et al. 2003), (Kiefer et al. 2022). In fact, monotherapy with SSRIs is not recommended but should be used in combination with psychotherapy, such as cognitive behavioral therapy (Kiefer et al. 2022). The British National Institute for Health and Care Excellence (NICE) guidelines also recommend benzodiazepines for assisted withdrawal therapies. They propose a fixed dose regime, which should only be adjusted in patients with severe withdrawal symptoms (NICE UK 2011).

Extensive literature addresses the pharmacological treatment options for AUD, most of which involve the use of drugs that are approved for AUD and aim to prevent relapse or achieve abstinence, such as disulfiram, acamprosate and naltrexone (Antonelli et al. 2022; Burnette et al. 2022; Kranzler 2023; Kranzler and Soyka 2018). Furthermore, a vast body of research analyzing the treatment of withdrawal syndrome is available (Antonelli et al. 2022; Ntais et al. 2005). However, studies evaluating the routine drug regimens used in clinical practice for AUD are scarce. While few studies have focused on outpatient treatment including pharmacotherapy (Muncie et al. 2013; Soyka and Müller 2017), research on inpatient populations is limited. Our study aims to fill this gap by analyzing data from various hospitals over a 16-year period. This cross-sectional analysis examines psychotropic drug therapy among psychiatric inpatients in German-speaking countries, outlines changes in prescribing practices over time, and assesses the treatment of concomitant diseases by analyzing patients’ comorbidities.

## Methods

The data for this study were collected by the *drug safety program in psychiatry* (German: “Arzneimittelsicherheit in der Psychiatrie”, AMSP). AMSP is a continuous multicenter drug surveillance program in which data on pharmacotherapy and severe adverse drug reactions in psychiatric inpatients have been collected since 1993. AMSP methods have been described in detail elsewhere (Grohmann et al. 2004). This program aims to improve the safety of pharmacological treatment in psychiatry. In 2016, 52 psychiatric institutions in Germany, Austria and Switzerland participated in the AMSP program. All participating hospitals gathered anonymous information about the use of psychotropic and nonpsychotropic drugs in all inpatients on two reference days each year including daily dosage of all drugs, primary psychiatric diagnosis and sex and age of the patients. As of 2007, secondary and tertiary psychiatric diagnoses were also documented. Psychiatric diagnoses were recorded by the treating psychiatrists according to the International Classification of Diseases (ICD-10 from 2000 to present). Evaluations based on AMSP data were approved by the Ethics Committee of the University of Munich and the Ethics Committee of the Hannover Medical School (Nr. 8100_BO_S_2018). This study adheres to the Declaration of Helsinki and its later amendments.

For our analysis, we used data from the AMSP database from 2000 to 2016 collected from hospitals in Germany (n = 69), Austria (n = 17) and Switzerland (n = 19). Patients who had a primary diagnosis of alcohol-related psychiatric and behavioral disorder diagnosis, i.e., ICD-10 F10.0-F10.9, were included in this study. We used the term AUD for better readability. The original dataset comprised data from 10,416 patients who received 38,870 prescriptions. Eighty-four patients with the diagnosis key F10.*, i.e., an unknown subclass of the F10, were removed. Demographics and drug utilization were analyzed in the remaining cohort.

Psychotropic drugs are classified into one of nine different types: antidepressant drugs, antipsychotic drugs, tranquilizing drugs (including benzodiazepines such as diazepam, lorazepam, oxazepam, alprazolam, bromazepam, clobazam, opipramol, herbal drugs), hypnotic drugs (including Z-drugs, benzodiazepines such as nitrazepam, midazolam, flunitrazepam, flurazepam, temazepam, herbal drugs, melantonine, antihistamines), anticonvulsant drugs, antiparkinsonian drugs, nootropics, lithium and “other” psychotropic drugs (e.g., clomethiazole). Antipsychotic drugs were further divided into high-potency and low-potency first-generation antipsychotics (FGAs) and second-generation antipsychotics (SGAs). The antidepressant drug subgroups included SSRIs, noradrenergic and specific serotonergic antidepressants (NaSSAs), serotonin noradrenaline reuptake inhibitors (SNRIs) and TCAs. We defined antidepressant drugs with other complex pharmacological mechanisms as *other antidepressant* drugs (e.g., bupropion, trazodone, agomelatine).

Dose equivalents were calculated based on the *defined daily dose* (DDD). This internationally recognized measure is available for most psychotropic drugs (Leucht et al. 2016).

To analyze the changes in psychotropic drug use over time, the dataset was divided into four time periods (2000–2004, 2005–2008, 2009–2012, and 2013–2016), with a similar number of patients in each group.

Categorical variables are displayed in frequency distribution tables. Cross-tabulations were used to investigate associations between two or more categorical variables, such as drug use, diagnosis, sex category and age group. The descriptive statistics used to analyze the categorical variables included frequencies and relative frequencies (i.e., frequencies divided by the population size, expressed as percentages). Continuous data (e.g., dose, DDD) were analyzed using measures of central tendency, i.e., arithmetic mean and median, and measures of dispersion, i.e., range and standard deviation. The χ^2^-test was used to calculate the statistical significance of differences between categorical variables. To compare the means of normally distributed continuous variables between two groups, Student’s t-test was used. A p-value > 0.05 indicates statistical significance.

## Results

The dataset included 10,332 patients with a primary diagnosis of AUD or acute intoxication with a total of 38,780 drug prescriptions. AUD inpatients comprised 7.3% of all 143,436 psychiatric inpatients assessed on the reference days in the study period.

The majority of AUD patients were male (69.4%) and aged 30–60 years (77.0%). The mean age was 48.6 (± 12.1) years. Males were slightly younger than females (48.1 ± 11.9 vs. 49.4 ± 12.6 years, (t(10,330) = *5.0621*, p < 0.001). The proportion of female patients was greater among patients aged ≥ 60 years (Table 1).

**Table 1:** Study population by age, sex, and primary diagnosis in subtypes of F10 according to the ICD-10

	< 31 years n (% of 739)	31–60 years n (% of 7990)	> 60 years n (% of 1603)	N and (%) of total 10,332 patients	Difference between male and female patients (χ^2^, p)
Female	220 (29.8)	2360 (29.5)	583 (36.4)	3163 (30.6)	
Male	519 (70.2)	5630 (70.5)	1,020 (63.6)	7169 (69.4)	
Primary diagnosis					
F10.0—acute intoxication	80 (10.8)	796 (10.0)	122 (7.6)	998 (9.7)	0.07, 0.80
Female	29 (3.9)	221 (2.8)	52 (3.2)	302 (2.9)	
Male	51 (6.9)	575 (7.2)	70 (4.4)	696 (6.7)	
F10.1—harmful use	80 (10.8)	271 (3.4)	70 (4.4)	421 (4.1)	**12.78, < 0.001**
Female	27 (3.7)	108 (1.4)	27 (1.7)	162 (1.6)	
Male	53 (7.2)	163 (2.0)	43 (2.7)	259 (2.5)	
F10.2—dependence syndrome	491 (66.4)	5406 (67.7)	906 (56.5)	6803 (65.8)	**10.03, 0.002**
Female	149 (20.2)	1661 (20.8)	343 (21.4)	2153 (20.8)	
Male	342 (46.3)	3745 (46.9)	563 (35.1)	4650 (45.0)	
F10.3—withdrawal state	69 (9.3)	955 (12.0)	138 (8.6)	1162 (11.2)	**35.13, < 0.001**
Female	12 (1.6)	213 (2.7)	43 (2.7)	268 (2.6)	
Male	57 (7.7)	742 (9.3)	95 (5.9)	894 (8.7)	
F10.4—withdrawal state with delirium	4 (0.5)	152 (1.9)	65 (4.1)	221 (2.1)	**11.17, < 0.001**
Female	0 (0.0)	32 (0.4)	13 (0.8)	45 (0.4)	
Male	4 (0.5)	120 (1.5)	52 (3.2)	176 (1.7)	
F10.5—psychotic disorder	10 (1.4)	69 (0.9)	34 (2.1)	113 (1.1)	0.08, 0.77
Female	3 (0.4)	25 (0.3)	8 (0.5)	36 (0.3)	
Male	7 (0.9)	44 (0.6)	26 (1.6)	77 (0.7)	
F10.6—amnesic syndrome	0 (0.0)	220 (2.8)	184 (11.5)	404 (3.9)	3.23, 0.07
Female	0 (0.0)	69 (0.9)	71 (4.4)	140 (1.4)	
Male	0 (0.0)	151 (1.9)	113 (7.0)	264 (2.6)	
F10.7—residual and late-onset psychotic disorder	1 (0.1)	93 (1.2)	71 (4.4)	165 (1.6)	0.59, 0.44
Female	0 (0.0)	22 (0.3)	24 (1.5)	46 (0.4)	
Male	1 (0.1)	71 (0.9)	47 (2.9)	119 (1.2)	
F10.8—other mental and behavioral disorders	3 (0.4)	13 (0.2)	8 (0.5)	24 (0.2)	0.36, 0.55
Female	0 (0.0)	4 (0.1)	2 (0.1)	6 (0.1)	
Male	3 (0.4)	9 (0.1)	6 (0.4)	18 (0.3)	
F10.9—unspecified mental and behavioral disorder	1 (0.1)	15 (0.2)	5 (0.3)	21 (0.2)	0.46, 0.50
Female	0 (0.0)	5 (0.1)	0 (0.0)	5 (0.0)	
Male	1 (0.1)	10 (0.1)	5 (0.3)	16 (0.2)	

Bold values indicate stastical significance p<0.05

The most common diagnosis was dependence syndrome (F10.2, 65.8%), followed by withdrawal states (F10.3, 11.2%) and acute intoxication (F10.0, 9.7%) (Table 1). The subdiagnoses F10.1, F10.2, F10.3 and F10.4 were significantly more common in men than in women (p < 0.05 each) (Table 1).

In young patients (< 31 years), subdiagnoses F10.0 and F10.1 were more common, while F10.4, F10.5, F10.6 and F10.7 affected older (> 60 years) patients more often (Table 1).

Data on secondary and tertiary psychiatric diagnoses were available for 6,968 AUD-patients for 2007–2016. Other substance use-related diagnoses (F1, 51.8%) and therein additional alcohol-related diagnoses (F10.x, 33.7%) were prevalent, followed by mood and affective disorders (F3, 18.4%) (Table 2). Only approximately one-third (34.6%) of patients did not have an additional psychiatric diagnosis*.* Almost half of the patients with a main diagnosis of acute intoxication had at least one secondary diagnosis of F10, and only 4% have no additional psychiatric diagnosis.

**Table 2:** Additional psychiatric diagnoses of alcohol use disorder patients in 2007–2016 (n = 6,968)

ICD-10-code	Additional diagnoses n (% of 6968)
No additional diagnosis	2410 (34.6)
F0 organic, including symptomatic, mental disorders	110 (1.6)
F1 mental and behavioral disorders due to psychoactive substance use	3,608 (51.8)
F10 due to use of alcohol	2347 (33.7)
F11 due to use of opioids	154 (2.2)
F12 due to use of cannabinoids	255 (3.7)
F13 due to use of sedatives or hypnotics	320 (4.6)
F14 due to use of cocaine	87 (1.2)
F15 due to use of other stimulants, including caffeine	40 (0.6)
F16 due to use of hallucinogens	5 (0.1)
F17 due to use of tobacco	287 (4.1)
F18 due to use of volatile solvents	4 (0.1)
F19 due to multiple drug use and use of other psychoactive substances	109 (1.6)
F2 schizophrenia, schizotypal and delusional disorders	217 (3.1)
F3 mood [affective] disorders	1280 (18.4)
F4 neurotic, stress-related and somatoform disorders	404 (5.8)
F5 behavioral syndromes associated with physiological disturbances and physical factors	34 (0.5)
F6 disorders of adult personality and behavior	197 (2.8)
F7 mental retardation	51 (0.7)
F8 disorders of psychological development	3 (0.0)
F9 unspecified mental disorder	47 (0.7)

### Drug utilization

Overall, 92.7% (n = 9,575) of patients were treated with at least one drug. Most patients were treated with psychotropic drugs (76.2%, n = 7,874) with an average of 1.9 ± 1.1 psychotropic drugs per patient. More women than men received psychotropic drugs (79.5% vs. 74.8%, p < 0.001). Among non-psychotropic drugs, vitamins, and proton pump inhibitors (PPIs) were used most frequently (42.2% and 28.4%, respectively).

Antidepressant (31.2%), antipsychotic (29.7%), anticonvulsant (26.4%) and tranquilizing drugs (24.3%) were frequently used, followed by other psychotropic (9.4%, mostly clomethiazole) and hypnotic drugs (5.3%). Antiparkinsonian drugs (2.6%), lithium (1.0%) and nootropic drugs (0.9%) were rarely applied (Table 3).

**Table 3:** Utilization of psychotropic drug groups over the period 2000–2016 by sex

	All patients n (% of 10,332)	Male patients n (% of 7169)	Female patients n (% of 3163)	Difference between male and female patients (χ2, p)
Any drug	9575 (92.7)	6599 (92.0)	2976 (94.1)	**13.44, < 0.001**
Any psychotropic drug	7874 (76.2)	5360 (74.8)	2514 (79.5)	**26.91, < 0.001**
Antidepressant drugs	3228 (31.2)	1904 (26.6)	1324 (41.9)	**239.17, < 0.001**
SSRI	1324 (12.8)	740 (10.3)	584 (18.5)	**130.20, < 0.001**
NaSSA	906 (8.8)	573 (8.0)	333 (10.5)	**17.63, < 0.001**
SNRI	631 (6.1)	334 (4.7)	297 (9.4)	**85.66, < 0.001**
TCA	560 (5.4)	345 (4.8)	215 (6.8)	**16.87, < 0.001**
Other AD	383 (3.7)	216 (3.0)	167 (5.3)	**31.59, < 0.001**
Antipsychotic drugs	3067 (29.7)	2050 (28.6)	1017 (32.2)	**13.31, < 0.001**
SGAs	1486 (14.4)	983 (13.7)	503 (15.9)	**8.55, 0.003**
Low-potency FGAs	1360 (13.2)	881 (12.3)	479 (15.1)	**15.65, < 0.001**
High-potency FGAs	720 (7.0)	521 (7.3)	199 (6.3)	3.22, 0.07
Anticonvulsant drugs	2731 (26.4)	1937 (27.0)	794 (25.1)	**4.14, 0.04**
Tranquilizing drugs	2515 (24.3)	1726 (24.1)	789 (24.9)	0.90, 0.34
Benzodiazepines	2443 (23,6)	1680 (23.4)	763 (24.1)	0.58, 0.45
Hypnotic drugs	547 (5.3)	334 (4.7)	213 (6.7)	**18.85, < 0.001**
Z-drugs	308 (3.0)	185 (2.6)	123 (3.9)	**13.00, < 0.001**
Benzodiazepines	121 (1.2)	77 (1.1)	44 (1.4)	1.91, 0.17
Herbal drugs	102 (1.0)	64 (0.9)	38 (1.2)	2.14, 0.14
Antiparkinson drugs	273 (2.6)	195 (2.7)	78 (2.5)	0.46, 0.50
Lithium	106 (1.0)	78 (1.0)	28 (0.9)	0.70, 0.40
Nootropic drugs	88 (0.9)	52 (0.7)	36 (1.1)	**3.95, 0.05**
Other psychotropic drugs	968 (9.4)	717 (10.0)	251 (7.9)	**11.03, < 0.001**
Clomethiazole	610 (5.9)	480 (6.7)	130 (4.1)	**26.41, < 0.001**
Acamprosat	223 (2.2)	145 (2.0)	78 (2.5)	2.04, 0.15
Disulfiram	96 (0.9)	63 (1.2)	33 (1.3)	0.65, 0.42

Significant results in bold

*SSRIs* selective serotonin reuptake inhibitors, *NaSSAs* noradrenergic and specific serotonin reuptake inhibitors, *SNRIs* serotonin noradrenaline reuptake inhibitors, *TCAs* tricyclic antidepressants, *AD* antidepressant drugs, *SGA* second generation antipsychotic drugs, *FGA* first generation antipsychotic drugs

Antidepressant, antipsychotic and hypnotic drugs were more commonly used in women, only anticonvulsant drugs were used more often in men. There was no difference in the use of tranquilizing drugs (Table 3).

Among antidepressant drugs, SSRIs (12.8%) and NaSSAs (8.5%) were most frequently used, followed by SNRIs, TCAs and other antidepressant drugs (6.1%, 5.4% and 3.7%, respectively). Among antipsychotic drugs, SGAs (14.4%) and low-potency FGAs (13.2%) were used in nearly equal proportions, while high-potency FGAs were used less frequently (7.0%) (Table 3). Benzodiazepines prevailed among tranquilizing drugs (23.6%), while Z-drugs were most common among hypnotics (3.0%).

Because states of withdrawal (F10.3), delirium (F10.4) and psychotic disorder (F10.5) associated with AUD tend to require distinct treatment, we performed a separate analysis of the drug utilization patterns of these diagnostic subgroups. Patients with F10.3, F10.4 and F10.5 received more drugs in general and more psychotropic drugs but considerably fewer antidepressant drugs than the total group of patients with AUD. All patients and in particular patients with F10.3 were less often treated with antipsychotic drugs than patients with F10.4 or F10.5. Tranquilizing drugs were given more often to patients with F10.3 and F10.4, while the use of hypnotic drugs was lower in these groups than in the whole population. The opposite was found among patients with F10.5. The utilization rate of hypnotic drugs was greater and that of tranquilizing drugs was lower than the rates for all patients, especially for patients with primary diagnoses of F10.3 and F10.4. Anticonvulsant drugs were given relatively often to patients with F10.3 and less often to patients with F10.5. One of the greatest differences was detected for clomethiazole, which was more commonly used for patients with F10.3 and F10.4 (Table 4).

**Table 4:** Comparison of the utilization of psychotropic drug groups by patients’ primary diagnosis (F10.3, F10.4 and F10.5)

	All patients n (% of 10,332)	F10.3 withdrawal state n (% of 1,163)	F10.4 withdrawal state with delirium n (% of 221)	F10.5 psychotic disorder n (% of 113)
Any drug	9,575 (92.7)	1,091 (93.8)	217 (98.2)	108 (95.6)
Any psychotropic drug	7,874 (76.2)	895 (77.0)	191 (86.4)	102 (90.3)
Antidepressant drugs	3,228 (41.0)	225 (25.1)	28 (14.7)	22 (21.6)
Antipsychotic drugs	3,067 (39.0)	280 (31.3)	125 (65.4)	89 (87.3)
Tranquilizing drugs	2,515 (31.9)	323 (36.1)	103 (53.9)	31 (30.4)
Anticonvulsant drugs	2,731 (34.7)	364 (40.7)	61 (31.9)	17 (16.7)
Hypnotic drugs	547 (6.9)	28 (3.1)	8 (4.2)	8 (7.8)
Antiparkinson drugs	273 (3.5)	63 (7.0)	10 (5.2)	5 (4.9)
Lithium	106 (1.3)	4 (0.4)	0 (0.0)	0 (0.0)
Nootropic drugs	88 (1.1)	3 (0.3)	4 (2.1)	0 (0.0)
Clomethiazole	610 (5.9)	119 (10.2)	29 (13.1)	1 (0.9)

Furthermore, we analyzed the utilization rates for patients with additional non-AUD psychiatric diagnoses (Table 5). For this, we divided patients into two groups: patients with at least one additional psychiatric diagnosis other than F10.x and patients without.

**Table 5:** Drug groups used for patients without and with additional psychiatric diagnoses from 2007 to 2016 (n = 6968)

2007–2016	All patients n (% of 6968)	Patients with only F10 diagnoses n (% of 3994)	Patients with other psychiatric diagnoses n (% of 2974)	χ^2^, p	Patients with additional diagnoses of F3 n (% of 1280)
Any drug	6599 (94.7)	3736 (93.5)	2863 (96.3)	**24.74, < 0.001**	1255 (98.0)
Any psychotropic drug	5410 (77.6)	2853 (71.4)	2557 (86.6)	**207.78, < 0.001**	1180 (92.2)
Antidepressant drugs	2332 (33.5)	817 (20.5)	1515 (50.9)	**710.14, < 0.001**	922 (72.0)
SSRI	921 (13.2)	316 (7.9)	605 (20.3)	**228.57, < 0.001**	375 (29.3)
NaSSA	674 (9.7)	249 (6.2)	425 (14.3)	**125.70, < 0.001**	274 (21.4)
SNRI	508 (7.3)	136 (3.4)	372 (12.5)	**207.66, < 0.001**	256 (20.0)
TCA	355 (5.1)	139 (3.5)	216 (7.3)	**49.67, < 0.001**	109 (8.5)
Other AD	325 (4.7)	105 (2.6)	220 (7.4)	**86.10, < 0.001**	131 (10.2)
Antipsychotic drugs	2200 (31.6)	1074 (26.9)	1126 (37.9)	**94.47, < 0.001**	454 (35.5)
SGAs	1160 (16.6)	460 (11.5)	700 (23.5)	**176.63, < 0.001**	261 (20.4)
Low-potency FGAs	945 (13.6)	461 (11.5)	484 (16.3)	**32.16, < 0.001**	224 (17.5)
High-potency FGAs	449 (6.4)	319 (8.0)	130 (4.4)	**36.37, < 0.001**	26 (2.0)
Tranquilizing drugs	1921 (27.6)	1068 (26.7)	853 (28.7)	3.12, 0.08	336 (26.3)
Benzodiazepines	1875 (26.9)	1051 (26.3)	824 (27.3)	1.68, 0.19	323 (25.2)
Anticonvulsant drugs	1755 (25.2)	1035 (25.9)	720 (24.2)	2.54, 0.12	301 (23.5)
Hypnotic drugs	339 (4.9)	143 (3.6)	196 (6.6)	**32.72, < 0.001**	90 (7.0)
Benzodiazepines	71 (1.0)	37 (0.9)	34 (1.1)	0.79, 0.37	17 (1.3)
Z-drugs	180 (2.6)	74 (1.9)	106 (3.6)	**19.84, < 0.001**	44 (3.4)
Antiparkinson drugs	171 (2.5)	97 (2.4)	74 (2.5)	0.01, 0.94	24 (1.9)
Lithium	68 (1.0)	27 (0.7)	41 (1.4)	**8.00, 0.01**	30 (2.3)
Nootropic drugs	37 (0.5)	23 (0.6)	14 (0.5)	0.19, 0.67	4 (0.3)

Significant results in bold

*SSRIs* selective serotonin reuptake inhibitors, *NaSSAs* noradrenergic and specific serotonin reuptake inhibitors, *SNRIs* serotonin noradrenaline reuptake inhibitors, *TCAs* tricyclic antidepressants, *AD* antidepressant drugs, *SGA* second generation antipsychotic drugs, *FGA* first generation antipsychotic drugs

Overall, patients with additional psychiatric diagnoses were treated with any kind of psychotropic drug more often than patients with only an F10 diagnosis (86.6% vs. 71.4%) We found significantly higher utilization rates for patients with additional psychiatric diagnoses than for patients without an additional psychiatric diagnosis for almost all kinds of drugs, with the exception of tranquilizing, anticonvulsant and antiparkinson drugs and nootropics. Anticonvulsant drugs were more commonly used for patients with only an F10 diagnosis than for patients with any other additional diagnoses, but the difference was not significant. Antidepressant drugs were given more than twice as often to patients with additional psychiatric diagnoses (50.9% vs. 20.5%).

We further analyzed differences in drug use according to age (i.e., ≤ 30, 31–60, > 60 years). Both the frequency of overall drug use and the use of psychotropic drugs increased with age. The proportion of patients receiving at least one psychotropic drug increased from 71.3% among those ≤ 30 to 77.2% among those > 60 years. A total of 27.9% of patients aged 31–60 years received an antipsychotic drug, while the frequency of using this group of drugs was greater among younger (< 31 years) and older (> 60 years) patients (36.4% and 35.7%, respectively). Tranquilizing and hypnotic drugs were given more frequently with increasing age (Table 6).

**Table 6:** Psychotropic drug groups from 2000–2016 by age

	< 30 n (% of 739)	31–60 n (% of 7990)	> 61 n (% of 1603)
Any drug	640 (86.6)	7372 (92.3)	1563 (97.5)
Any psychotropic drug	527 (71.3)	6110 (76.5)	1237 (77.2)
Antidepressant drugs	213 (28.8)	2525 (31.5)	490 (30.6)
Antipsychotic drugs	269 (36.4)	2226 (27.9)	572 (35.7)
Tranquilizing drugs	137 (18.5)	1952 (24.4)	426 (26.6)
Anticonvulsant drugs	165 (22.3)	2212 (27.7)	354 (22.1)
Hypnotic drugs	36 (4.9)	398 (5.0)	113 (7.0)

### Drugs and dosages

Regarding the use of individual drugs, carbamazepine was the most common drug (11.1%), followed by diazepam (10.1%), mirtazapine (8.5%) and oxazepam (8.2%) (Table 7).

**Table 7:** Most frequently used drugs and their median dose and mean DDD (daily defined dose)

Drugs	n (% of 10,332)	Mean DDD (SD)	Median (min/max) in mg
Carbamazepine	1143 (11.1)	0.5 (0.2)	600 (15/1600)
Diazepam	1046 (10.1)	1.9 (1.6)	15 (0,5/240)
Mirtazapine	883 (8.5)	1.0 (0.6)	30 (1/300)
Oxazepam	845 (8.2)	1.2 (1.2)	40 (1,5/400)
Quetiapine	658 (6.4)	0.5 (0.5)	125 (12,5/1500)
Clomethiazole	610 (5.9)	0.6 (1.2)	576 (0/38400)
Haloperidol	553 (5.4)	0.6 (1.1)	5 (0,5/75)
Lorazepam	525 (5.1)	1.2 (1.4)	2 (0,25/60)
Venlafaxine	475 (4.6)	1.7 (0.8)	150 (37.5/450)
Valproate	456 (4.4)	1.2 (0.6)	1000 (20/3000)
Citalopram	402 (3.9)	1.5 (2.7)	20 (2/1000)
Oxcarbazepine	402 (3.9)	0.7 (0.3)	600 (150/2100)
Escitalopram	398 (3.9)	1.4 (0.6)	10 (2.5/40)
Pipamperone	376 (3.6)	0.3 (0.2)	40 (10/260)
Risperidone	372 (3.6)	0.6 (1.1)	2 (0.5/100)
Prothipendyl	305 (3.0)	0.3 (0.1)	80 (40/280)
Olanzapine	293 (2.8)	1.4 (0.8)	10 (1/75)
Sertraline	292 (2.8)	1.7 (0.9)	75 (10/200)
Doxepin	272 (2.6)	0.9 (0.7)	75 (6,25/600)
Trazodone	262 (2.6)	0.5 (0.2)	150 (25/450)

Commonly used antipsychotic drugs were quetiapine (6.4%) and haloperidol (5.4%). Clomethiazole was given to 5.8% of all patients. Lorazepam was more commonly used in women (women: 7.1% vs. men: 4.2%, p < 0.001), while diazepam was given more often to men (men: 10.9% vs. women: 8.3%, p < 0.005). The biggest differences between the sexes were found for the antidepressant drugs venlafaxine (women: 7.1% vs. men: 3.5%, p < 0.001), escitalopram (women: 5.8% vs. men: 3.0%, p < 0.001), citalopram (women: 5.5% vs. men: 3.2%, p < 0.001) and trazodone (women: 4.0% vs. men: 1.0%, p < 0.001) and the antipsychotic drug quetiapine (women: 8.3% vs. men: 5.5%, p < 0.001), all of which were given to women more often. On the other hand, men were treated more often with clomethiazole (men: 6.7% vs. women: 4.1%, p < 0.001) (Table S1).

Drugs to support relapse prevention were used in 3.5% of all patients, mainly acamprosate (2.2%), followed by disulfiram (0.9%), naltrexone (0.4%), and nalmefen (0.1%).

As described above, we analyzed drug use in patients with additional psychiatric diagnoses for all drugs used in ≥ 2.5% of patients. Among patients with additional psychiatric diagnoses, mirtazapine (14.2%) and quetiapine (12.7%) were the most commonly used drugs, whereas mirtazapine (6.2%) and quetiapine (5.4%) were used less often in patients without additional psychiatric diagnoses. The use of diazepam and oxazepam was similar in both groups (11.9% vs. 11.4%), 9.4% vs. 10.3% resp.). Antidepressant drugs were given more often to patients with additional psychiatric diagnoses than to those without, e.g., venlafaxine (9.0% vs. 2.4%), escitalopram (6.8% vs. 2.9%), citalopram (5.7% vs. 2.5%) and sertraline (5.0% vs. 1.8%). Anticonvulsant drugs such as carbamazepine (6.3% vs. 10.6%) and oxcarbazepine (3.0% vs. 4.6%) were more often used in patients without additional psychiatric diagnoses. Clomethiazole was given approximately twice as often to patients with only AUD (6.2% vs. 3.3%). Furthermore, haloperidol was the only antipsychotic drug that was more commonly used in patients without additional psychiatric diagnoses (7.0% vs. 2.8%). In contrast, all other antipsychotic drugs were used more often in patients with additional psychiatric diagnoses (Table S2).

Next, we calculated the average prescribed dose in relation to the drug’s DDD, which represents its assumed average maintenance dose per day for its primary indication in adults. We found mean dose equivalents significantly exceeding one DDD for diazepam (1.9 ± 1.6 times DDD, median dose 15 mg/day), venlafaxine (1.7 ± 0.8 times DDD, median dose 150 mg/day), and sertraline (1.7 ± 0.9 times DDD, median dose 75 mg/day). In contrast, relatively low dose equivalents were observed for quetiapine (0.5 ± 0.5 times DDD, median dose 125 mg/day), pipamperone (0.3 ± 0.2 times DDD, median dose 40 mg/day), carbamazepine (0.5 ± 0.2 times DDD, median dose 600 mg/day), prothipendyl (0.3 ± 0.1 times DDD, median dose 80 mg/day), and trazodone (0.5 ± 0.2 times DDD, median dose 150 mg/day) (Table 7).

### Time trends

**Fig. 1:**
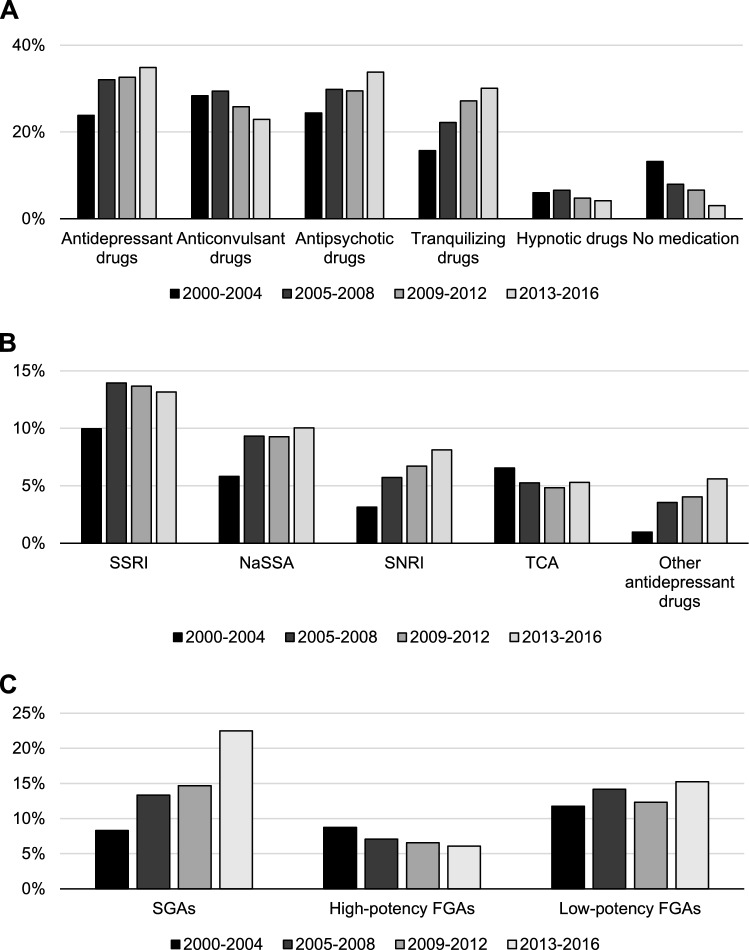
Changes in the utilization spectrum of psychoactive drugs in 4–5-year periods from 2000 to 2016: **A** main classes of psychotropic drugs, **B** antidepressant drugs, **C** antipsychotic drugs. N: 2000–2004: 2168, 2005–2008: 2533, 2009–2012: 2774, 2013–2016: 2857

The use rates of antidepressant (23.8% to 34.9%) and antipsychotic drugs (24.4% to 33.8%) increased, and the use of tranquilizing drugs nearly doubled from the first (2000–2004: 15.7%) to the last period (2013–2016: 30.1%) (Fig. 1). Anticonvulsant (28.4% to 22.9%) and hypnotic drugs (6.0% to 4.2%) as well as the proportion of patients without any drugs (13.2% to 3.0%) decreased (Fig. 1A).

Both the average number of drugs per patient (3.5 to 4.3) and the average number of psychotropic drugs per patient (1.8 to 2.0) increased over time. With regard to age, we found no significant differences or trends in the number of psychotropic drugs. Among the non-psychotropic drugs, the utilization rates for PPIs increased drastically from the first (2000–2004: 13.2%) to the last period (2013–2016: 39.0%). Additionally, vitamins were more frequently used (35.1% to 48.9%).

Among antidepressant drugs, the use of SNRIs (3.1% to 8.1%), NaSSAs (5.8% to 10.0%) and “other antidepressant drugs” (1.0% to 5.6%) increased steeply from 2000–2004 to 2013–2016, primarily due to the increasing use of venlafaxine (3.1% to 6.0%), mirtazapine (5.2% to 10.0%) and trazodone (0.6% to 3.7%). While the use of citalopram remained stable (3.0% and 2.9%), the use of its enantiomer escitalopram increased from 1.1% in the first period to 4.1% in the last period. The only decreasing utilization rate among the antidepressant drugs was found for TCAs (6.5% to 5.3%) (Fig. 1B).

The large increase in the use of antipsychotic drugs is mainly caused by the increasing utilization rate of SGAs (8.3% to 22.5%) and therein quetiapine (0.9% to 11.9%). The use of low-potency FGAs increased as well (11.8% to 15.3%) (Fig. 1C), among which pipamperone (3.0% to 4.7%) and prothipendyl (1.3% to 3.5%) were used more often.

Tranquilizing drug utilization rates increased over time. For example, oxazepam rates increased from 4.2% to 11.4%, and diazepam rates nearly doubled from the first to the last period (6.6% to 13.0%).

The utilization of hypnotic drugs decreased slightly. In this group, the benzodiazepine analogues zolpidem and zopiclone were given most often, although their use decreased from 2000–2004 to 2013–2016 (zopiclone 2.2% to 1.1%; zolpidem 1.8% to 1.0%).

The utilization rates of carbamazepine (17.5% to 6.1%) and clomethiazole (8.3% to 3.3%) decreased steeply from the first to the last period.

## Discussion

We analyzed the patient and drug use data of 10,332 psychiatric inpatients in German-speaking countries with a diagnosis of AUD or acute intoxication between 2000 and 2016. AUD patients represented 7,3% of all psychiatric inpatients. While this appears low, many AUD patients remain untreated often due to poor insight or willingness to undergo the necessary therapie (Schaller et al. 2022). Furthermore, the present study only included inpatients with a primary diagnosis of AUD. Many more may have had AUD as a secondary diagnosis and would therefore not have been included in the present evaluation.

We found a greater percentage of male patients, which is consistent with previous reports (Schaller et al. 2022; Van Der Linde et al. 2014). Men are not only at greater risk of developing AUD, but also more prone to suffering from a more severe course.

In our study, almost two-thirds of AUD patients were diagnosed with dependence syndrome, followed by withdrawal state and acute intoxication. Schaller et al. reported comparable findings but with higher rates of patients with acute intoxication, likely due to the inclusion of all medical specialties versus only psychiatric inpatients as in the present study. Regarding age, they reported similar patterns. Younger patients were mostly hospitalized with acute intoxication, while dependence syndrome was the most common admission diagnosis among older patients. They also found that women were hospitalized more often with F10.2 than with other subdiagnoses (Schaller et al. 2022).

AUD typically develops after prolonged heavy drinking, increasing the risk of addiction and secondary health complications in older patients (Rehm et al. 2017), as was seen in the present study with an increase in severe psychiatric alcohol-related complications such as withdrawal state with delirium, alcohol-induced psychotic disorder and alcohol-related amnesic syndrome with age.

We found that drug use varied depending on the primary AUD diagnosis. Clomethiazole was most frequently used to treat F10.4, in line with guidelines that recommend its use in combination with antipsychotic drugs (Kiefer et al. 2022). Also consistent with guideline recommendations was that haloperidol was a commonly used antipsychotic drug in our study (Kiefer et al. 2022). It is favored not only for managing acute psychotic symptoms but also for states of agitation or aggressive behavior, which may account for the high use of haloperidol in young men who may be more prone for this type of behavior. This could explain the high utilization of haloperidol not only in older adults but also in younger patients. Furthermore, as opposed to other antipsychotic drugs such as risperidone, haloperidol is not used off-label.

The treatment of AUD in the inpatient setting is presumably guided by withdrawal symptoms and the prevention of severe complications such as seizures, which may explain the use of anticonvulsant and tranquilizing drugs. While sleeping disorders and restlessness are common in patients suffering from addiction, they may be even more prevalent in patients during withdrawal (Koob and Colrain 2020). Therefore, drugs with sleep-inducing effects, such as low-potency antipsychotics, TCAs, mirtazapine, and quetiapine, may be helpful and explain their high utilization in the present study.

In this study, both the proportion of inpatients treated with psychotropic drugs and the average number of psychotropic drugs per patient increased over time, consistent with previous AMSP data on other mental disorders (Greiner et al. 2020). Although the overall utilization rates for psychotropic drugs (76.2%) were lower than those for other patient groups in comparable AMSP studies– e.g., major depression disorder (93.4% to 98.7%) (Seifert et al. 2021a, b, c), borderline personality disorder (90.0%) (Bridler et al. 2015) and disorders due to sedative-hypnotics (95.6%) (Pauwels et al. 2025)—it remains substantial. Patients solely diagnosed with only AUD received fewer psychotropic drugs compared to those with additional psychiatric diagnoses (71.4% vs. 86.6%). Patients with AUD suffering from comorbid psychiatric diagnoses often experience severe health impairments and have less favorable prognoses (Kiefer et al. 2022). For this reason, the guidelines explicitly emphasize the importance of properly addressing comorbidities and providing appropriate treatment (Kiefer et al. 2022), (NICE UK 2011). This could be even more a reason for the more frequent use of drugs, which are not approved for the treatment of AUD but for the treatment of those comorbidities.

As previously described by others (Grant et al. 2004), mood disorders – especially depressive disorders – were common in our cohort (18.4%), at least partly accounting for the impressive utilization rates of antidepressant drugs. SSRIs in particular are recommended for the treatment of comorbid depression in patients with AUD (Kiefer et al. 2022). Among patients with comorbid depression in this study, antidepressant drugs were given more than three times as often as to patients with only AUD. We found a decreasing utilization rate of TCAs over time, although not to the extent that was reported in other studies using AMSP data, for example, among patients with major depressive disorder (Seifert et al. 2021a, b, c). In the present study, doxepin was the most commonly used TCA, and its use remained stable during the observation period (approximately 2.6%). This may be due to its approved use for the treatment of mild to moderate withdrawal syndromes. Apart from SSRIs, the older version of the German guideline for the treatment of patients with AUD recommends the utilization of TCAs or SSRIs for comorbid depression (Mundle et al. 2003). The newer version, however, no longer mentions the use of TCAs to treat comorbid depression, and SSRIs are not recommended as monotherapy (Kiefer et al. 2022). Of note, our data refer to a time before the new version, which was published in 2020 and updated in 2022. The NICE guideline explicitly points out that antidepressant drugs should not be used for AUD therapy alone (NICE UK 2011).

We found increasing utilization rates of tranquilizing drugs, while the use of anticonvulsant drugs decreased. Furthermore, the use of clomethiazole decreased from 8.3% (in 2000–2004) to 3.3% (2013–2016). Interestingly, this trend was first observed before the new guidelines were published, suggesting that clinical practice preceded guideline recommendations (Mundle et al. 2003), (Kiefer et al. 2022). While clomethiazole was initially recommended as a first-line therapy (Mundle et al. 2003), benzodiazepines are now preferred—when used for a limited time—due to their favorable drug safety profile (Kiefer et al. 2022). Benzodiazepines are recommended for both the management of severe withdrawal symptoms and the prevention of seizures (Kiefer et al. 2022), suggesting that the use of tranquilizing drugs appropriately replaced the use of anticonvulsant drugs, as observed in this study. In addition, the NICE guidelines state that benzodiazepines should be used only for managing withdrawal symptoms and not for the ongoing treatment of alcohol dependence (NICE UK 2011).

Our study found that the use of tranquilizing drugs increases with patient age, a trend that may have multiple contributing factors. First, older patients are more likely to have experienced multiple withdrawal episodes, increasing their risk for severe withdrawal symptoms and complications. This, in turn, necessitates a greater use of tranquilizing drugs to effectively manage their symptoms (Goodson et al. 2014). The treatment for these patients should ideally involve benzodiazepines —particularly those with a short half-life and minimal liver metabolism, such as oxazepam (Kiefer et al. 2022).

We found that antipsychotic drugs were frequently used to treat patients with AUD, and their use has increased over time. The German guidelines recommend treatment with antipsychotic drugs only for delirium and psychotic symptoms and only in combination with clomethiazole or benzodiazepines (Kiefer et al. 2022). Our analysis showed that the increased use of antipsychotic drugs is mostly due to the utilization of the SGA quetiapine. The average dose of 125 mg implies that quetiapine was primarily used for its sedative and not for its antipsychotic properties. The use of quetiapine may appear favorable in comparison to that of other drugs (e.g., benzodiazepines, Z-drugs) because quetiapine does not carry a risk for dependence and appears to have satisfactory tolerability. Although in the meantime, the potential for abuse, especially in connection with alcohol and other substance abuse, has been noted several times (Chiappini and Schifano 2018; Malekshahi et al. 2015; Vento et al. 2020). Furthermore, it is worth noting that quetiapine is not approved for the treatment of AUD, deeming its use in this indication “off-label”. While it appears to be common practice in our naturalistic patient setting, Maher et al. (Maher and Theodore 2012) state that evidence does not support the use of atypical antipsychotics such as quetiapine in treating substance use disorders. Among all patients treated with quetiapine in our study, 63.5% had an additional psychiatric diagnosis, with a particularly high percentage of patients with personality and behavioral disorders (F60.*: 12.6%).

The present study revealed very low use of drugs for relapse prevention. One reason for this may be the inpatient setting of this study, whereas the German guidelines recommend these drugs specifically for the outpatient treatment of patients with repeated relapses (Kiefer et al. 2022). The NICE-Guideline proposes the use of drugs to prevent relapse in patients with moderate and severe alcohol dependences after successful withdrawal only in combination with an individual psychological intervention (NICE UK 2011). Within this drug group, acamprosate was the most commonly used drug and was used to treat 2.2% of all patients in our study. This suggests that treatment with these drugs may be initiated during an inpatient stay to prepare patients for the ambulatory setting. Meta-analytical data demonstrate the effectiveness of naltrexone and acamprosate for maintaining abstinence (Donoghue et al. 2015; Petrov et al. 2011) but not for returning to abstinence after drinking relapse (Szabo and Lippai 2014).

Notably, in addition to the increasing use of psychotropic drugs, we also observed a greater utilization of non-psychotropic drugs (e.g., vitamins, PPIs). Patients with AUD often suffer from physical consequences and diseases resulting from long-term alcohol misuse, such as malnutrition which can lead to severe and potentially life-threatening vitamin deficiencies. Therefore, vitamin supplementation—particularly vitamin B1 (thiamine)—is imperative to help prevent Wernicke’s encephalopathy (Galvin et al. 2010; Szabo and Lippai 2014).

Similarly, PPI use increased over time in our patient sample. Among individuals with AUD, PPIs are commonly used due to the high prevalence of gastric conditions such as gastroesophageal reflux disease and the increased risk of ulcers, gastrointestinal bleeding and carcinoma (Haber and Kortt 2021). However, the necessity of PPIs should be regularly reassessed. Both chronic PPI use and chronic alcohol abuse contribute to an increased risk of osteoporosis and fractures and their combined effects can significantly worsen bone health (Kellerman and Kintanar 2017). Furthermore, PPIs may elevate the risk of hyponatremia, particularly when combined with psychotropic medications such as SSRIs and SNRIs. Since patients with AUD may already be more susceptible to hyponatremia, this interaction could have an even greater impact in this population (Seifert et al. 2021a, b, c).

We found several differences in the utilization of psychotropic drugs between the sexes. Due to the paucity of relevant studies on sex-specific pharmacotherapy for any psychiatric disease, we can only speculate about the reasons. Psychosocial differences in the effects of alcohol between men and women have been described: while women are more likely to suffer from mood and anxiety disorders, the prevalence of narcissistic and antisocial personality disorders is greater among men (Goldstein et al. 2012). Additionally, drinking motives, especially for the first use of alcohol, differ between the sexes (Substance Abuse and Mental Health Services Administration 2014). Men tend to seek disinhibition, potentially resulting in risky behavior, while women prefer anxiolytic and relaxing effects. Oliva et al. reported that women were more likely to report anxiety and depression at the beginning and at the end of hospitalization for alcohol detoxification than men (Oliva et al. 2018). This may explain the greater use of drugs with anxiolytic effects (e.g., lorazepam, citalopram, escitalopram) in women.

We found a greater utilization of antidepressant drugs in women with AUD, which has previously also been reported for inpatients and outpatients with major depressive disorder (Seifert et al. 2021a, b, c), (Andersson Sundell et al. 2011). Both the NICE and the German S3 guidelines for depression suggest that women may benefit more from treatment with SSRIs, whereas men tend to benefit more from TCAs (Cleare et al. 2015; Deutsche Gesellschaft Für Psychiatrie and Ärztliches Zentrum Für Qualität In Der Medizin (ÄZQ) 2017). A greater use of SSRIs in women was also found in our study, while TCAs were also more commonly used in women than in men. This observation was also made by Seifert et al. in a study on inpatients with major depressive disorder using AMSP data (Seifert et al. 2021a, b, c).

Moreover, Soya et al. reported a greater frequency of severe complications of withdrawal syndrome including seizures in men than in women (Soyka et al. 2006; Unlu et al. 2023). This may explain the greater use of anticonvulsant drugs in men.

### Limitations

Several limitations should be considered when interpreting the results of our study. Because the collected data and reported diagnoses are based on routine clinical data, certain aspects, such as interfering somatic diagnoses,were not assessed in our data. Furthermore, it is not possible to differentiate which medications were newly administered and which had already been taken by the patients before and were continued. AMSP is unable to provide data regarding relevant clinical aspects of treatment (e.g., course of clinical symptoms, nonpharmacological treatment). Drug use and polypharmacy might be overestimated due to cross-tapering strategies. Because drug utilization may vary between hospitals and countries, changes in the participation of different hospitals during the observation period may partly explain changes in drug use patterns. Furthermore, it cannot be excluded that any changes observed in this study were due to changing patient cohorts within hospitals. In general, cross-sectional data do not allow conclusions to be drawn about causal relationships.

## Conclusion

We found a relatively high rate of psychotropic drug use (76.2%) in patients with AUD, even in those without comorbid mental health disorders. Our data suggest that increasing attention should be given to comorbidities and their treatment. While the use of certain drugs is indicated for the management of acute withdrawal symptoms (e.g., intermittent use of benzodiazepines) and secondary diagnoses (e.g., antidepressant drugs), we found high utilization rates of several drugs with sedative properties, such as trazodone, mirtazapine, and quetiapine. Although common in practice, there is insufficient evidence to support their use in the treatment of AUD, indicating that there is still a need for studies on the effect of these drugs on withdrawal and abstinence maintenance. Nonetheless, our results demonstrate a relatively high adherence to guideline recommendations. In fact, our data suggest that the utilization patterns we observed (e.g., decreasing use of clomethiazole and increasing use of benzodiazepines) even preceded the guideline recommendations, which were revised after the end of our study period.

## Supplementary Information

Below is the link to the electronic supplementary material.

Supplementary file1 (PDF 162 KB)

## Data Availability

The dataset was obtained from the anonymized database of AMSP and cannot be accessed online.

## Abbreviations

AUD: Alcohol use disorder
AMSP: Arzneimittelsicherheit in der Psychiatrie
ICD: International Classification of Diseases
DGPPN: Deutsche Gesellschaft für Psychiatrie, Psychotherapie, Psychosomatik und Nervenheilkunde
NICE: National Institute for Health and Care Excellence
TCA: Tricyclic antidepressant drug
SSRI: Selective serotonin reuptake inhibitor
FGA: First-generation antipsychotic drug
SGA: Second-generation antipsychotic drug
NaSSA: Noradrenergic and specific serotonergic antidepressant drug
SNRI: Serotonin noradrenaline reuptake inhibitor
DDD: Defined daily dose
AD: Antidepressant drug
PPI: Proton pump inhibitor
SD: Standard deviation
