# Supplementary material for: Pharmacotherapy for psychiatric inpatients with alcohol use disorder or acute intoxication: results from an observational pharmacovigilance program—status and changes between 2000 and 2016
Source: J Neural Transm (Vienna). 2025 Sep 4;133(3):547–60. doi: 10.1007/s00702-025-03012-z (PMC12999762; doi:10.1007/s00702-025-03012-z)
Supplement: Supplementary file 1 — Supplementary file1 (PDF 162 KB) [file 702_2025_3012_MOESM1_ESM.pdf]

Supplement to

Pharmacotherapy for psychiatric inpatients with alcohol use disorder: Results from an observational pharmacovigilance program - status and changes between 2000 and 2016

Submission for Journal of Neural Transmission

Authors: Beatrice Haack[1] (beatrice.haack@mhb-fontane.de), Johanna Engel[1], Philipp Pauwels[1], Sermin Toto[2], Stefan Bleich[2], Johanna Seifert[2], Renate Grohmann[3], Martin Heinze[1], Oliver Zolk[4], Phileas Proskynitopoulos[2], Timo Greiner[1], Michael Schneider[1]

Affiliations:

[1]University Department of Psychiatry and Psychotherapy, Brandenburg Medical School, Immanuel Klinik Rüdersdorf, 15562 Rüdersdorf, Germany

[2]Department of Psychiatry, Social Psychiatry and Psychotherapy, Hannover Medical School, 30625 Hannover, Germany

[3]Department of Psychiatry and Psychotherapy, LMU University Hospital, LMU Munich, 80336 Munich, Germany

[4]Institute of Clinical Pharmacology of the Brandenburg Medical School, Immanuel Klinik Rüdersdorf, 15562 Rüdersdorf, Germany

Table S1: Most frequently used drugs by sex (n>2.5%)

Drug	All patients n (% of 10,332)	Male patients n (% of 7,169)	Female patients n (% of 3,163)	Difference between male and female patient (χ^2, p)
Carbamazepine	1,143 (11.1)	847 (11.8)	296 (9.4)	**13.46, < 0.001**
Diazepam	1,046 (10.1)	782 (10.9)	264 (8.3)	**15.83, < 0.001**
Mirtazapine	883 (8.5)	558 (7.8)	325 (10.3)	**17.43, < 0.001**
Oxazepam	845 (8.2)	584 (8.1)	26 (8.3)	0.03, 0.86
Quetiapine	658 (6.4)	394 (5.5)	264 (8.3)	**29.91, < 0.001**
Clomethiazole	610 (5.9)	480 (6.7)	130 (4.1)	**26.41, < 0.001**
Haloperidol	553 (5.4)	422 (5.9)	131 (4.1)	**13.19, < 0.001**
Lorazepam	525 (5.1)	301 (4.2)	224 (7.1)	**37.83, < 0.001**
Venlafaxine	475 (4.6)	252 (3.5)	223 (7.1)	**62.53, < 0.001**
Valproate	456 (4.4)	332 (4.6)	124 (3.9)	2.63, 0.11
Citalopram	402 (3.9)	229 (3.2)	173 (5.5)	**30.38, < 0.001**
Oxcarbazepine	402 (3.9)	290 (4.0)	112 (3.5)	1.49, 0.22
Escitalopram	398 (3.9)	216 (3.0)	182 (5.8)	**44.52, < 0.001**
Pipamperone	376 (3.6)	247 (3.4)	129 (4.1)	2.51, 0.11

Risperidone	372 (3.6)	260 (3.6)	112 (3.5)	0.05, 0.83
Prothipendyl	305 (3.0)	190 (2.7)	115 (3.6)	**7.44, 0.006**
Olanzapine	293 (2.8)	215 (3.0)	78 (2.5)	2.26, 0.13
Sertraline	292 (2.8)	182 (2.5)	110 (3.5)	**7.05, 0.008**
Doxepin	272(2.6)	187(2.6)	85 (2.7)	0.05, 0.82
Trazodone	262(2.5)	134(1.9)	128 (4.0)	**42.11, < 0.001**

significant results in bold

Table S2: Most frequently used drugs for patients without and with additional psychiatric diagnoses from 2007 to 2016 (n =6968)

Drug	Patients with additional psychiatric diagnoses n (% of 3,994)	Patients with only F10 diagnosis n (% of 2,974)
Mirtazapine	422 (14.2)	247 (6.2)
Quetiapine	377 (12.7)	217 (5.4)
Diazepam	354 (11.9)	457 (11.4)
Oxazepam	279 (9.4)	413 (10.3)
Venlafaxine	269 (9.0)	96 (2.4)
Escitalopram	201 (6.8)	115 (2.9)
Carbamazepine	188 (6.3)	423 (10.6)
Lorazepam	188 (6.3)	180 (4.5)
Citalopram	171 (5.7)	98 (2.5)
Valproate	153 (5.1)	137 (3.4)
Pipamperone	150 (5.0)	131 (3.3)
Sertraline	148 (5.0)	73 (1.8)
Trazodone	145 (4.9)	76 (1.9)
Olanzapine	132 (4.4)	61 (1.5)
Risperidone	119 (4.0)	134 (3.4)
Duloxetine	100 (3.4)	33 (0.8)
Doxepin	100 (3.4)	74 (1.9)
Clomethiazole	97 (3.3)	246 (6.2)
Pregabalin	95 (3.2)	58 (1.5)
Prothipendyl	95 (3.2)	132 (3.3)
Oxcarbazepine	88 (3.0)	183 (4.6)
Haloperidol	84 (2.8)	281 (7.0)
Chlorprothixene	81 (2.7)	40 (1.0)
Melperone	81 (2.7)	93 (2.3)
Clonazepam	79 (2.7)	47 (1.2)
Promethazine	77 (2.6)	66 (1.7)
Acamprosate	73 (2.5)	66 (1.7)
